# 454 sequencing reveals extreme complexity of the class II Major Histocompatibility Complex in the collared flycatcher

**DOI:** 10.1186/1471-2148-10-395

**Published:** 2010-12-31

**Authors:** Magdalena Zagalska-Neubauer, Wiesław Babik, Michał Stuglik, Lars Gustafsson, Mariusz Cichoń, Jacek Radwan

**Affiliations:** 1 Institute of Environmental Sciences, Jagiellonian University, Gronostajowa 7, 30-387 Kraków, Poland; 2 Ornithological Station, Museum and Institute of Zoology, Polish Academy of Sciences, Nadwiślańska 108, 80-680 Gdańsk, Poland; 3 Department of Ecology and Genetics/Animal Ecology, Evolutionary Biology Centre, Uppsala University, Norbyvägen 18D, S-752 36 Uppsala, Sweden

## Abstract

**Background:**

Because of their functional significance, the Major Histocompatibility Complex (MHC) class I and II genes have been the subject of continuous interest in the fields of ecology, evolution and conservation. In some vertebrate groups MHC consists of multiple loci with similar alleles; therefore, the multiple loci must be genotyped simultaneously. In such complex systems, understanding of the evolutionary patterns and their causes has been limited due to challenges posed by genotyping.

**Results:**

Here we used 454 amplicon sequencing to characterize MHC class IIB exon 2 variation in the collared flycatcher, an important organism in evolutionary and immuno-ecological studies. On the basis of over 152,000 sequencing reads we identified 194 putative alleles in 237 individuals. We found an extreme complexity of the MHC class IIB in the collared flycatchers, with our estimates pointing to the presence of at least nine expressed loci and a large, though difficult to estimate precisely, number of pseudogene loci. Many similar alleles occurred in the pseudogenes indicating either a series of recent duplications or extensive concerted evolution. The expressed alleles showed unambiguous signals of historical selection and the occurrence of apparent interlocus exchange of alleles. Placing the collared flycatcher's MHC sequences in the context of passerine diversity revealed transspecific MHC class II evolution within the Muscicapidae family.

**Conclusions:**

454 amplicon sequencing is an effective tool for advancing our understanding of the MHC class II structure and evolutionary patterns in Passeriformes. We found a highly dynamic pattern of evolution of MHC class IIB genes with strong signals of selection and pronounced sequence divergence in expressed genes, in contrast to the apparent sequence homogenization in pseudogenes. We show that next generation sequencing offers a universal, affordable method for the characterization and, in perspective, genotyping of MHC systems of virtually any complexity.

## Background

The Major Histocompatibility Complex (MHC) includes the most polymorphic genes in vertebrates [1]. MHC polymorphism is functionally relevant and is thought to be maintained by selection through the mechanisms of heterozygote advantage and frequency dependence, driven by the host-pathogen interactions and, in some cases, disassortative mating preferences (reviewed in [2]). The products of the MHC genes are involved in triggering the adaptive immune response against pathogens [1], and may also play a role in mate choice and individual recognition (reviewed in [3,4]). Because of their functional significance, MHC class I and II genes have been the subject of continuous interest in the fields of ecology, evolution and conservation (reviewed in [2,3,5,6]).

Usually, multiple MHC class I and II loci are present in a given species. In some species, genes within each class are both structurally and functionally divergent [7]. However, in other groups multiple loci may contain similar (and thus presumably more or less functionally equivalent) alleles, or identical alleles may even be shared among loci [8,9]. Repeated expansions and contractions of the number of MHC loci observed in several vertebrate groups are thought to result from frequent duplications followed by a birth and death process [10-12]. In some taxa, concerted evolution through gene conversion appears to be important, and recombination may generate additional variation [13-16]. These processes may be manifested at the population level by the coexistence of haplotypes that differ in the number of loci [17,18]. A comprehensive understanding of the actual mechanisms that generate and maintain copy number variation in the MHC requires substantial population genomic information from multiple species.

A well-established consequence of the presence of multiple loci containing similar or identical alleles and of copy number variation is a relatively broad range of per-individual number of alleles within a population. It has been proposed that in such situations, selection may favor individuals with an intermediate number of alleles (reviewed in [3]). This may be due to the tradeoff between the ability to present the maximum number of pathogen antigens, which is positively correlated with the number of MHC alleles, and the ability to invoke an effective immune response, which may be negatively correlated with individual MHC diversity because a higher proportion of lymphocytes must be eliminated to prevent immune autoaggression problems [19,20]. Data from sticklebacks, sparrows and bank voles appear to support the optimality hypothesis [21-24]. However, the hypothesis remains controversial [25,26] and, for its thorough evaluation, additional data from natural populations of species showing extensive variation in individual MHC diversity is necessary.

MHC structure differs substantially between bird species, even within the same family [27]. Some species, such as chicken or parrots, have an extremely small, compact MHC, dubbed "the minimal essential MHC" [28,29]. Other birds, such as owls [30], exhibit more complex MHC structure with multiple loci that may retain orthologous relationships over long periods of evolutionary time, a situation resembling the patterns observed in mammals [7,12]. The MHC of passerine birds is very complex, commonly consisting of multiple expressed loci and pseudogenes [14,31-36]. The relationships among passerine MHC genes tend to mirror phylogeny, with sequences of multiple loci generally grouped according to species, indicating that extensive sequence exchange among loci occurs in these birds [9,14]. Repeated rounds of duplication and homogenization via gene conversion or exon shuffling among genes [8,14] are thought to be responsible for this pattern. The approximate number of MHC loci in passerines has been estimated through Southern Blot experiments followed by limited cloning and sequencing [33,37]. Although this approach readily distinguishes between simple and complex systems, and may provide information about the relationships among loci, it is not well suited for studying the patterns of sequence variation in multilocus systems. A comprehensive characterization of such complex systems, which is necessary for the understanding of their evolution, requires extensive sampling of sequences from individuals and across multiple individuals. Genomic data on the MHC organization, which provide precise estimates of the number of MHC loci (although, typically, do not allow to assess among-individual variation) have been obtained so far only for one passerine species - the zebra finch [36].

Because of the presence of multiple MHC loci with generally similar alleles, which may probably be regarded as functionally equivalent, passerine birds are an ideal system for studying the relationship between the strength of immune response, mate choice and individual MHC diversity, as measured by the number and the sequence divergence of MHC alleles. However, an efficient and reliable genotyping method is a prerequisite for such studies.

The presence of multiple, often similar alleles that commonly cannot be assigned to loci and must be genotyped simultaneously poses substantial methodological challenges [38]. None of the methods that have been widely used in the field offer straightforward, reliable and accurate genotyping that is scalable to systems of arbitrary complexity [38]. The advent of next generation sequencing (NGS) technologies (reviewed in [39]) has brought the promise of such a method. Indeed one of the NGS technologies, 454 pyrosequencing, has already been successfully applied for genotyping complex multilocus MHC systems [24,40]. Reliability of 454 genotyping in multilocus was verified by cloning [24] and by replicate genotyping [24,41]. Next generation sequencing technologies have several key advantages for MHC genotyping. They are equivalent to cloning single-stranded DNA molecules derived from amplicons in a cell-free system, thus they avoid artifacts commonly observed during cloning in biological vectors and propagation in bacteria [42,43]. Parallel sequencing of clonally amplified templates produces hundreds of thousands of sequencing reads and does not require colony picking, clone handling and Sanger sequencing, the costly and time-consuming procedures that have limited the throughput of traditional cloning approaches. With NGS, the coverage of several hundreds or thousands of sequencing reads per amplicon can be achieved at a moderate cost of a few euro per sample, massive multiplexing allowing simultaneous analysis of hundreds or thousands of samples in a single analysis is possible, and analyses can be completed within days. The major problem with applying NGS technologies to MHC genotyping appears distinguishing true alleles from various kinds of artifacts [40,41]. However this problem is common to all PCR-based methods [38].

Here, we used 454 pyrosequencing to assess the MHC class IIB variation in the collared flycatcher with two major goals in mind. First, we used the massive amount of sequence data produced in our experiment to advance the understanding of the evolutionary patterns and processes occurring in the passerine MHC. Specifically, we were interested in: i) estimating the number of MHC class IIB loci, with special emphasis on expressed loci, ii) comparing patterns of diversity among expressed and pseudogene sequences to infer the mechanisms driving their evolution and iii) evaluating the relationship of the collared flycatcher MHC class IIB sequences with the MHC II of other Passerine species. Second, we aimed to establish a foundation for genotyping the MHC in a model organism for evolutionary and immuno-ecological studies [44-46]. Interpretation of the large body of data regarding parasite load, immune response and mating preferences in this species can greatly benefit from an immunogenetic perspective.

## Results

### Sequencing

A part of the exon 2 of the MHC class II B gene was amplified and sequenced for 237 collared flycatchers; 39 individuals were amplified twice with different tags and two individuals were amplified three times. Thus, a total of 280 amplicons were sequenced. We obtained 152,053 188-bp sequences with complete tags. Only 35 tagged primers were used for amplification, however theoretically any of 4,096 possible 6-bp tag sequences could have been generated via errors in primer synthesis and/or sequencing. We obtained 502 reads with tags that did not match any of the used tag sequences; these reads comprised 0.33% of all reads. To estimate the maximum level of misassignment of reads to amplicons, we needed to consider the proportion of unused tags which was 99.1%. Therefore, the maximum level of misassignment was approximately equal to the fraction of reads with tags not used in the amplification primers, i.e. 0.33%. Although the estimated level of misassignment was about two times higher than reported previously [40,47], it was still low enough to ensure that misassigned reads are rare, and as such will not affect the outcome of genotyping, which requires an allele to be present in multiple reads. Moreover, tags differed from each other by at least three substitutions, which reduces the chance of misassignment although does not eliminate it entirely, because of indel mutations.

The mean coverage (number of reads per amplicon) was 541 ± (SD) 166 and ranged from 88 (an apparent outlier, as the next lowest coverage was 182) to 1022. Taking into account the misassignment rate given above, we expected an average of less than two misassigned reads per amplicon, but given sequence differences between used tags, probably even less. Among 20,060 unique sequences, there were 13,927 singletons (9.2% of all reads) and 6,133 variants represented by at least two reads.

#### Sequence diversity and expression status

**Figure 1:**
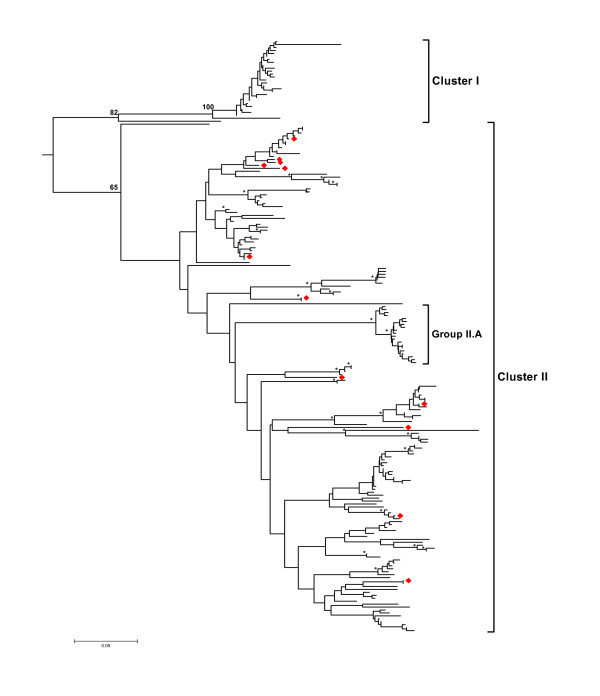
**Relationship among the MHC class II alleles**. Neighbor Joining tree showing relationships among the collared flycatcher MHC class II putative alleles. Sequences obtained from cDNA are marked red. The tree was constructed from the matrix of Tamura-Nei nucleotide distances and rooted with the chicken sequence (not shown). The robustness of the tree was tested with 1000 bootstrap replicates, bootstrap supports for major clades are indicated with numbers, and other bootstrap values higher than 70% are marked with asterisks.

In this study, 194 sequence variants fulfilled the criteria for distinguishing true alleles from artifacts resulting from PCR (base substitutions and chimeras) and 454 sequencing (substitutions and indels) and are further considered as putative alleles (PA). Cloning and Sanger sequencing of cDNA from three additional individuals revealed twelve alleles (adding seven new variants to the list). Sequences of all variants were deposited in GenBank (accession numbers: HQ678311-HQ678511) The relationships among PA are shown in Figure 1. Inspection of the tree reveals the presence of two clusters (I and II), each with a considerable number of PA. However, the PA in these clusters show dissimilar patterns of divergence. While most PA in cluster I are extremely similar to each other, commonly differing by only one substitution (Figure 1), cluster II PA are on average much more divergent, forming multiple lineages that are considerably different from each other. The relationships among these lineages are poorly resolved. A group of 20 very similar PA (group II.A) stands out among other cluster II sequences. Group II.A is strongly supported, with 100% bootstrap support and is separated by a long branch from other cluster II PA.

The pronounced differences in the degree of divergence among PA in various parts of the tree may indicate that these variants experienced different evolutionary histories. A likely explanation for the dissimilar patterns is that multiple evolutionary mechanisms played a predominant role in shaping the diversity of sequences depicted in Figure 1. Important insights in this respect are provided by an analysis of expression. All sequence variants obtained from cDNAs by cloning and Sanger sequencing fell into cluster II. Five of twelve variants supported by at least two clones were identical to PA obtained in the 454 run. None of the expressed sequences fell into cluster I or into group II.A. Most of the cluster I PA contained a 1 or 2-bp deletion in the middle of exon 2, causing a frameshift. Putative alleles in group II.A contained two deletions, 9-bp and 1-bp, also resulting in a frameshift. Taken together, these observations constitute strong evidence that cluster I and group II.A PA represent nonfunctional pseudogenes. Two observations suggest that most of the sequences in cluster II (excluding group II.A) may be functional MHC class IIB alleles. First, sequences obtained from cDNA are widely distributed across the tree, falling into many divergent lineages (Figure 1). Second, no frameshift mutations or stop codons occur in any sequences.

It should be noted here, that, due to higher sequence similarity among cluster I pseudogenes compared to cluster II putative expressed alleles (PEA), our criteria for distinguishing artifacts from true alleles (detailed in Methods) were more conservative for cluster I pseudogenes than for PEA. This is because, if many sequences differ by only 1-2 substitutions, many of them can be interpreted as chimeras between other very similar sequences. The consequences of this is illustrated by the fact that from 157 cluster II PEA which passed the preliminary 2-PCRs-3-copies-in-each criterion, 146 (93.0%) were retained after the PCR chimera filtering step. On the contrary, from 225 cluster I pseudogene sequences only 28 (12.4%) were retained. Thus, many more of true cluster I pseudogene alleles than the number we estimated are certainly present in the collared flycatchers.

An inspection of the sequences of PAs indicated that for the studies requiring efficient genotyping of functional MHC class IIB variation, the coverage required may be considerably reduced by preventing amplification of the cluster I sequences by extending the forward primer by one base pair (G) in the 3' direction, since all cluster II sequences contain a G in this position, whereas all cluster I sequences have a C or T.

### Estimating the number of MHC class IIB loci

The maximum number of PEA per individual provides an estimate of the number of expressed MHC class IIB loci in the collared flycatcher. Counting only PEA present in at least two reads per amplicon, the maximum number of alleles per individual was 18 (mean = 9.6 ± (SD) 3.2). It is possible however, that some of variants present in any given individual are in fact PCR chimeras identical to PEA. To evaluate this possibility we checked genotypes of all nine amplicons with the highest number of PEAs (16-18), and in each amplicon discarded all PEAs which could have been classified as PCR chimeras of more abundant (as measured by the number of reads) PEAs. In four amplicons there were no such variants, in two aplicons one variant, in two amplicons two variants, and in one amplicon three possible PCR chimeras were present. It should be noted here, that these variants were not necessarily PCR chimeras, because true recombinant alleles may co-occur with both parental sequences in some individuals. Thus, excluding all such cases gives a conservative estimate of the maximum number of PEA per individual at 17 alleles, corresponding to at least nine expressed loci.

Estimation of the maximum number of pseudogene loci is more difficult for two reasons. First, as explained in the previous section, a substantial and difficult to estimate number of true pseudogene alleles were excluded from the analysis because they could not be reliably distinguished from PCR chimaeras on the level of the full dataset. Second, again due to a very high sequence similarity among pseudogene alleles, on the individual level, true pseudogene alleles may often be explained as chimeras, therefore an analysis similar to this performed for PEA in the above paragraph would not reflect any biological reality. Signalling these serious limitations imposed by the nature of the data, we do not want to leave the reader without an idea about the maximum number of pseudogene loci. Therefore we present maximum numbers based on at least two reads for amplicon, similarly as these for PEA. The maximum number of cluster I pseudogene alleles was 19 (mean = 12.7, SD = 2.4) and that of the group II.A was 9 (mean = 2.64, SD = 1.3). These estimates thus point to the presence of at least 10 cluster I and at least 5 group II.A pseudogene loci in the collared flycatcher.

Replicate genotypes, derived from two or three independent amplicons, were obtained for 41 individuals. On average 24.6 PA were present in both (or all three in case of triplicated samples) replicates (at least one read in each), and 4.6 in only one replicate (a PA must have been present in at least two reads in one replicate and in no reads in the second replicate). The respective values for PEA were 9.6 and 2.5, for cluster I 12.4 and 0.4 and for group II.A 2.4 and 0.4. A relatively high number of alleles, which were not confirmed across replicates, again indicate that coverage was not sufficient for replicate genotyping, the effect was particularly strong for PEA, as expected. As PEA constituted on average 16% of reads, slightly less than 90 reads of PEA per amplicon were obtained on average, which is not sufficient for reliable genotyping of even 4 loci (the maximum allowed by program) according to the criteria of Galan et al. [41] based on an idealized model assuming equal amplification of all alleles. Thus this coverage is even more inadequate for nine loci estimated in the present paper.

### Signatures of natural selection and recombination

**Figure 2:**
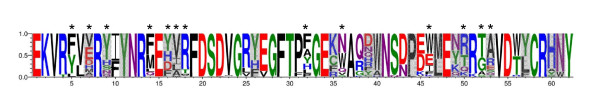
**Amino acid positions under positive selection**. A sequence logo showing the relative frequencies of various amino acids in particular positions of the examined fragment of the MHC class IIB 2^nd ^exon. The plot is based on sequences of all putative expressed alleles. Antigen Binding Sites (ABS) are shaded, and positions under positive selection as revealed by the Bayes Empirical Bayes (BEB) procedure are indicated with asterisks.

Codon-based tests of natural selection were performed separately for putative expressed alleles, cluster I pseudogenes and group II.A pseudogenes (Tables 1 and 2). In order to keep the open reading frame, pseudogene sequences required the removal of some alignment columns and sequences exhibiting internal stop codons. In cluster II, for both putative expressed alleles and group II.A pseudogenes, the model of codon evolution allowing for positive selection produced a much better fit to the data than one dN/dS ratio (M0) or nearly neutral (M7) models (Table 1). However, only the expressed alleles showed a highly significant excess of nonsynonymous substitutions in the putative Antigen Binding Sites (ABS), but not in non-ABS, and extreme nonsynonymous divergence in ABS (Table 2). In the expressed alleles, the Bayes Empirical Bayes procedure identified thirteen codons as evolving under positive selection (Figure 2, all posterior probabilities (PP) ≥ 0.98). Six of these were located in ABS, the proportion of positively selected codons did not differ between ABS and non-ABS (*P *= 0.16, Fisher's exact test). An excess of nonsynonymous substitutions in either ABS or non-ABS was not observed for pseudogene sequences (Table 2). The two positively-selected codons detected in the group II.A pseudogenes were not located in ABS. For cluster I pseudogenes, the nearly neutral model of codon evolution (M7) fitted the data best (Table 2). All three methods detected recombination in the representative set of 25 sequences. The GARD method detected recombination at a *P *level of 0.01 in all ten datasets of 25 randomly selected sequences, whereas the Geneconv and MaxChi2 methods detected recombination in four datasets. Overall, recombination appears frequently in the collared flycatcher's MHC II, as it is easily detected in relatively small subsets of the data.

**Table 1:** Evaluation of the goodness of fit for different models of codon evolution and estimated parameter values

Model	lnL	ΔAIC	Parameters
	**Cluster I putative pseudogenes**		
	
M0 - one ω	-525.2	7.0	ω = 0.568
M7 - nearly neutral with beta	-520.7	best	
M8 - positive selection with beta (ω_0 _≤ 1, ω_1 _> 1)	-520.3	3.2	*p*_0 _= 0.925, *p*_1 _= 0.075, ω_1 _= 3.133
	**Cluster II putative expressed alleles**		
	
M0 - one ω	-4108.8	931.4	ω = 0.687
M7 - nearly neutral with beta	-3744.3	118.2	
M8 - positive selection with beta (ω_0 _≤ 1, ω_1 _> 1)	-3685.6	best	*p*_0 _= 0.748, *p*_1 _= 0.252, ω_1 _= 3.238
	**Group II.A (putative pseudogenes)**		
	
M0 - one ω	-353.5	48.0	ω = 2.332
M7 - nearly neutral with beta	-343.1	29.2	
M8 - positive selection with beta (ω_0 _≤ 1, ω_1 _> 1)	-326.5	best	*p*_0 _= 0.962, *p*_1 _= 0.038, ω_1 _= 50.098

ω - dN/dS; nearly neutral with beta - for all sites ω ≤ 1 and the beta distribution approximates ω variation; positive selection - a proportion of sites evolves with ω > 1; *p*_0 _- proportion of sites with ω ≤ 1, *p*_1 _- proportion of positively selected sites (ω > 1), ω_1 _- estimated value of ω for sites under positive selection; ΔAIC - the difference between the value of the Akaike Information Criterion (AIC) of a given model and the best model.

**Table 2:** Synonymous and nonsynonymous rates

Sites	dN	dS	*Z*	*P*
	**Cluster I putative psudogenes**			
	
**All**	0.033(0.007)	0.029(0.008)	0.37	0.71
**ABS**	0.029(0.011)	0.047(0.029)	-0.64	0.52
**non-ABS**	0.035(0.009)	0.024(0.010)	0.91	0.36
	**Cluster II putative expressed alleles**			
	
**All**	0.210(0.032)	0.119(0.032)	1.97	0.051
**ABS**	0.455(0.081)	0.128(0.054)	3.30	0.001*
**non-ABS**	0.139(0.033)	0.117(0.035)	0.55	0.58
	**Group II.A - putative pseudogenes**			
	
**All**	0.021(0.009)	0.007(0.008)	1.29	0.20
**ABS**	0.019(0.013)	0.000(0.000)	1.65	0.10
**non-ABS**	0.022(0.010)	0.009(0.008)	0.95	0.34

The average rates of nonsynonymous substitutions per nonsynonymous site (dN) and synonymous substitutions per synonymous sites (dS) computed according to the Nei-Gojobori method, with standard errors obtained through 1000 bootstrap replicates in parentheses, and the results of the *Z *test of neutrality. * denotes significant *P*.

### MHC class IIB of the collared flycatcher in the context of Passerine sequence diversity

**Figure 3:**
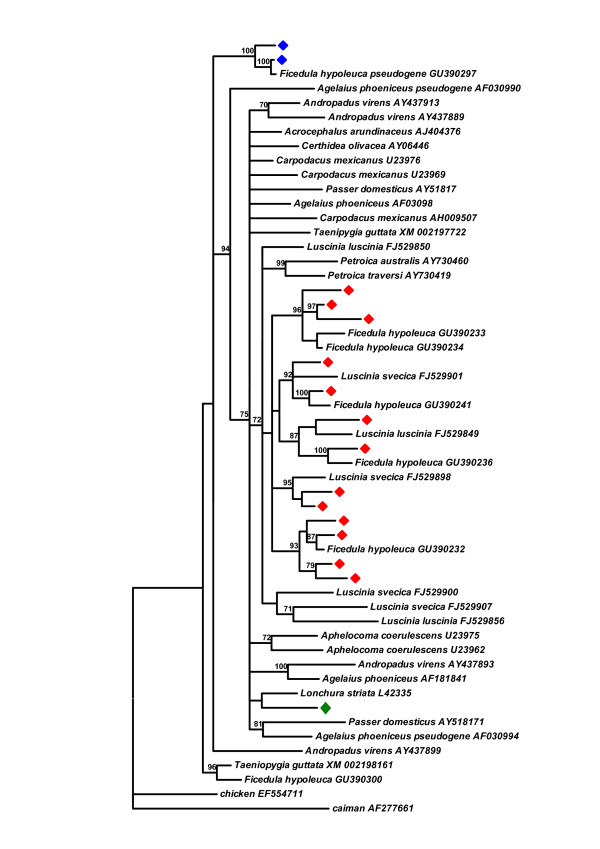
**Collared flycatcher MHC II in the context of diversity in Passeriformes**. Collared flycatcher sequences, representative of the entire MHC II diversity, in the context of MHC II diversity in Passeriformes. Collared flycatcher putative expressed alleles - red, putative pseudogene cluster I alleles - blue, putative pseudogene group II.A alleles - green. The majority rule consensus tree from the Bayesian analysis is shown with posterior probabilities (PP) at least 0.70 indicated; for clarity PP are presented as percentages. GenBank accession numbers are given next to the species names.

The tree in Figure 3 shows the relationship of a representative set (selected to encompass the entire MHC diversity) of the collared flycatcher's MHC class IIB sequences to other Passeriformes. The collared flycatcher's PEA fall into a single moderately supported clade (PP of 0.72). All expressed sequences from classical MHC class IIB loci available for the pied flycatcher (*Ficedula hypoleuca*), the collared flycatcher's sister species [48] grouped together with cluster II PEA. In fact, of 25 the pied flycatcher alleles four had identical sequences to the collared flycatcher's alleles. Several bluethroat and nightingale alleles fall into this cluster as well, with some of them forming highly supported (PP > 0.9) smaller clusters with collared flycatcher alleles. Although expressed alleles appear to form a clade, their diversity is comparable to the overall diversity of the available Passeriformes sequences. Both pseudogene lineages fall outside the expressed cluster, which may indicate their long independent evolutionary history. Interestingly the pseudogene sequence reported for the pied flycatcher was identical to one of the cluster I pseudogene alleles reported in the present study.

## Discussion

We demonstrated an extreme complexity of the MHC class IIB in the collared flycatchers, our estimates point to the presence of at least nine expressed and fifteen pseudogene loci, but this latter number is highly uncertain and likely to be a gross underestimate of the actual number of pseudogene loci. Hence, it is not surprising that the average coverage of 541 sequencing reads per amplicon was not sufficient for reliable genotyping. The massive amount of sequence data, generated in our study from the large population sample was, however, more than sufficient for assessing sequence diversity, contrasting mechanisms driving the evolution of expressed and pseudogene sequences, and advancing our understanding of the MHC class II structure and evolution in Passeriformes.

The biggest challenge in using 454 pyrosequencing for genotyping complex MHC systems is distinguishing true alleles from sequence artifacts that may emerge during PCR or sequencing. Some of these artifacts may be produced repeatedly, depending on the sequence context, particularly small insertions and deletions in regions with homopolymer runs [49,50]. Chimeras may also be generated repeatedly during independent PCRs via in vitro recombination between true alleles. It has been demonstrated that, given sufficient coverage, it is possible to establish frequency thresholds to effectively distinguish true alleles from artifacts. Replicate genotyping of a fraction of individuals from independently obtained amplicons is the natural choice for establishing genotyping thresholds [24,40]. For applications requiring exceptionally low genotyping error rates replicate genotyping of all samples may be desirable, and may be easily attained at a moderate cost [51]. However, identical chimaeras may occur in replicates, so an extra caution is needed when chimeras are expected to pose a serious problem. PCR protocols are available which should minimize chimera frequency [43]. The most important factor here appears the reduction of the number of PCR cycles, but keeping the number of PCR cycles at the low limit may lead to variation in DNA concentration among amplicons, low PCR success rate, limiting amounts of PCR product etc. When working with a large number of samples with DNA of variable quality, all these factors may impose a considerable logistical burden while not guaranteeing elimination of the chimera problem. Therefore it may be desirable to consider post-sequencing approaches for chimera elimination similar to the one outlined in the present paper.

In the present study, we did not achieve coverage sufficient for genotyping, but apparently we were able to eliminate most PCR and sequencing artifacts and obtained a comprehensive picture of the sequence diversity, at least for expressed loci, on the population level. The number of the putative expressed alleles present in the population and the lower bound for the maximum number of expressed loci we report should be close to reality, although may slightly underestimate the actual numbers due to a limited coverage of the PEA which constituted on average only ca 16% of reads per amplicon.

On the contrary, several lines of evidence indicate that we have only scratched the surface of the cluster I pseudogene diversity. First, cluster I pseudogenes represented on average 72% of reads per amplicon. If our primers amplify various sequence variants with similar efficiency, then the proportion of reads from a locus should roughly correspond to the number of copies of the locus in the genome. Sequences of 28 pied flycatcher's 2^nd ^exon alleles [48] span the binding sites of both PCR primers used in the present study. These sequences show excellent match with our primers ensuring efficient amplification of PEA. Comparison of the proportion of reads from the PEA, to the proportion of reads from cluster I pseudogenes may point to the existence of ca. 40 cluster I pseudogene loci in the collared flycatcher. Second, applying our conservative procedure for chimera elimination to variants identified under the 2-PCRs-3-copies-in-each criterion reduced the number of cluster I pseudogene variants considered as true alleles by almost 90% compared to a modest 7% reduction for the PEA.

Because of the extreme similarity of cluster I pseudogene alleles, estimation of the per individual number of loci may be challenging or even impossible using PCR-based techniques, because of chimera formation and the relatively high frequency of base-substitution errors. Possibly target-enrichment techniques coupled with next generation sequencing technologies, approaches that minimizes the use of PCR [52] could be helpful here.

MHC class IIB in the collared flycatcher is comprised of both putative expressed loci and pseudogenes. Only expressed alleles exhibited an excess of nonsynonymous substitutions and codons under positive selection in the putative Antigen Binding Sites, as expected for functional MHC sequences [2,6]. Pseudogene alleles showed signatures of nonfunctionality: frameshift-causing indels and the presence of internal stop codons in multiple sequences. Although codon-based tests of selection detected some signatures of positive selection in group II.A pseudogene sequences, the evidence was more ambiguous than in the case of expressed loci. Because signatures of positive selection may be retained for very long periods of evolutionary time [53], these inconsistent results of selection tests probably reflect ancient pseudogenization of once functional MHC sequences. MHC class IIB pseudogenes, also ancient, which diverged from functional loci over 40 Ma, have been described in a few other passerine species [32,54,55].

Sequences from expressed and pseudogene clusters differ not only in their signals of historical selection. A remarkable difference was also observed in the degree of divergence among expressed and pseudogene sequences. Most pseudogene alleles were very similar to each other (Figure 1), despite originating from a number of loci. Two mechanisms may generate extreme similarity of allele sequences among loci. Either pseudogenes have recently undergone series of duplications, or they have been evolving in concert through a mechanism of interlocus gene conversion. Recent duplications have been described in passerine MHC [55] and gene conversion is thought to be a major mechanism acting in the MHC of birds [33,56,57] although its importance has not yet been suggested for MHC pseudogene evolution. Distinguishing between these two mechanisms requires more extensive genomic information.

Among expressed alleles, both similar and highly divergent alleles occurred. However, these do not form well-supported, divergent lineages that can be interpreted as corresponding to different loci. Therefore, extensive genetic exchange among loci must have occurred, a conclusion also supported by the frequent cases of recombination detected among divergent sequences. Yet, this process has not homogenized allele sequences in a comparable way to that observed for pseudogenes. It is likely that the divergence has been maintained by selection which favored certain types of genetic exchange between loci such as reciprocal recombination shuffling entire exons, e.g. through recombination in introns [34] or gene conversion involving only short stretches of sequences [58]. Examining other parts of genes, such as exon 3 [30] or the 3' untranslated regions [8,57], may allow the identification of locus specific sequences. In principle, identification of locus-specific primers may be possible in introns flanking the 2^nd ^exon [58]. This approach was used in the pied flycatcher and although intron sequences discriminate pseudogenes and nonclassical MHC II B loci from classical class IIB loci, they do not allow locus-specific amplification of the latter [48]. The processes of duplication and interlocus genetic exchange, particularly gene conversion, may have operated synergistically to generate the patterns observed in the collared flycatcher. Segmental duplications could have boosted gene conversion, as it is known that physical proximity in the genome facilitates this process [59]. An interesting observation comes from the genomic analysis of MHC class II region in the zebra finch, where Balakrishnan et al. [36] found a large number of retroelements which may facilitate gene duplication in passerine MHC II.

Regardless of the details of recombination among expressed loci, the consequence of a high rate of interlocus recombination is a blurring of orthologous relationships and the grouping of alleles in a species-specific manner to the exclusion of other passerine sequences. The grouping of MHC class II sequences according to species and not gene phylogeny, is common among passerine birds [9,14,31]. Transspecific polymorphism is usually detected only among closely related passerine taxa [56,57], although exceptions are known [60]. The Bayesian tree, which places the collared flycatcher's MHC class IIB PA in the context of passerine MHC diversity, provides some support for grouping all collared flycatcher PEA together. These PEA are, however, intermixed with the MHC class IIB sequences of three other species from the Muscicapidae family, the pied flycatcher [48], the bluethroat and nightingale [35], and form well-supported clusters with them in a few cases (Figure 3) or even show identical sequences as some pied flycatcher's alleles. Thus, there is evidence for transspecific polymorphism in the Muscicapidae family not only at the genus level [35], but also among genera.

Even conservative estimates of the number of MHC class IIB loci located in collared flycatchers and bluethroats [35] place them among vertebrate species with the highest number of loci. It remains to be seen whether Muscicapidae are an exception among passerines, or whether, as other studies, based on RFLP, probe hybridization as well as cloning seem to suggest, possessing tens of MHC class IIB loci is the rule in these birds [9,61]. The zebra finch, the only passerine species sequenced so far has four expressed MHC class IIB loci and five pseudogenes [36], as inferred from the genome assembly; however there also appears to be variation in the number of loci among individuals as revealed by Southern Blot experiments in this species. Counting the actual number of loci has been notoriously difficult without the availability of genome sequences, and rough estimates of copy number for highly multiplicated MHC I genes have been obtained from blotting experiments [62,63]. Next generation sequencing will enable better characterization of copy number variation and allow the study of the relationships among paralog sequences, which is necessary for understanding the evolutionary and population genetic processes that shape their evolution. In any case, an extraordinary number of expressed MHC loci makes the collared flycatcher an excellent model system for testing hypotheses relating to individual MHC diversity, effectiveness of the immune response and mate choice. In this context, it is interesting to try to estimate the sequencing effort needed for reliable genotyping. We suggest that reliable genotyping of pseudogenes may require methods which minimize the use of PCR. Extrapolating the results from bank voles, which have at least six loci, where a mean coverage of 200-400 (mean 344) reads per amplicon was sufficient for reliable genotyping of MHC II [24], a coverage of 400-800 reads per amplicon should be sufficient for reliable genotyping of expressed loci in collared flycatchers. The actual coverage may need to be increased because of the co-amplification of the group II.A pseudogenes. Through the examination of sequences of hundreds putative alleles, we identified primers that can amplify all expressed alleles, and preliminary experiments indicate that these primers indeed amplify mostly PEA. Thus, we have laid a foundation for experimental studies of the relationships between individual MHC diversity, immune response and mate choice in this extremely interesting system.

## Conclusions

We found a highly dynamic pattern of evolution of MHC class IIB genes with strong signals of selection and pronounced sequence divergence in expressed genes, in contrast to the apparent sequence homogenization in pseudogenes. Our study has broad implications for testing hypotheses relating the individual MHC diversity, effectiveness of the immune response and mate choice. MHC genotyping in species particularly suited for rigorous evaluation of such hypotheses, i.e. showing multiple MHC loci as well as among-haplotype variation in the number of loci, has been notoriously difficult since appropriate methods have not been available. We showed that next generation sequencing is bringing the promise of overcoming this obstacle to progress in the field by offering a universal, affordable method for the characterization and in perspective genotyping of MHC systems of virtually any complexity.

## Methods

### Samples

We analyzed 237 females of the collared flycatcher *Ficedula albicollis *from the Gotland (Sweden) nest-box breeding population. For detailed description of the study area and species see [64]. Here we make use of samples obtained from females caught during breeding while incubating eggs or feeding young in year 2003. Birds were individually marked with standard aluminum rings ensuring that unique birds were used in the analyses. Blood was collected and stored in 96% ethanol. DNA extraction was performed with the NucleoSpin^® ^Blood kit (Macherey-Nagel).

### Development of primers

**Figure 4:**
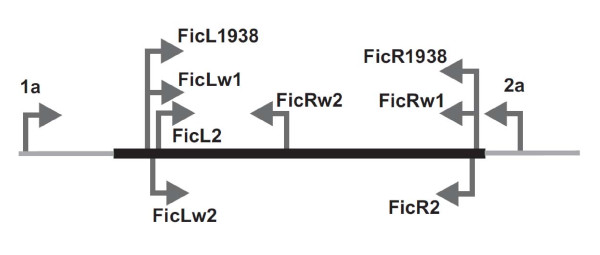
**Location of primers used in the study**. Location of primers on the MHC class IIB 2^nd ^exon is shown. Arrow heads are pointing in primers' 3' direction. Location of the 5' end of each primer is indicated.

To design primers amplifying a substantial part of the MHC class IIB exon 2 in the collared flycatchers we used two complementary approaches. First, we used conserved primers 1a and 2a [34] as well as primers FicL2/FicR2 (for sequences and location of all primers used in the study see Table 3 and Figure 4) located in highly conserved parts of the exon, identified from the alignment of passerine sequences available from the NCBI (Accession Numbers: AF030992, AJ404374, AY437903, AY518183, AY730452), to generate partial exon 2 sequences. We then used the consensus from these partial sequences to design specific primers within the exon (FicLw1 and FicLw2 in the 3' direction and FicRw1, and FicRw2 in the 5' direction), which led to successful amplification, through vectorette PCR, of longer 2^nd ^exon sequences covering the regions where we intended to place primers used for genotyping.

**Table 3:** Sequences of primers used in the study

Primer	Sequence (5'-3')
1a [34]	ATGGGACCCCAAAAGTGATT
2a [34]	CCGAGGGGACACGCTCT
FicL2	CTTCATTAACGGCACGGAGA
FicR2	GCGCTCCACGAGGAAC
FicLw1	GAGTGTCWCTTCDTTAACGGC
FicLw2	GTCACTTCDTTAACGGCACSGAGA
FicRw1	TCTGCGCTCCACGVKGAACGG
FicRw2	CGWACCGCCCCACGTCGCTGTCG
FicL1938	GAGTGTCHYTTCVTTAACGGCAC
FicR1938	CTCTGCGCTCCACGVBGAACGGG

In the vectorette PCR approach, total genomic DNA is digested with a restriction enzyme (RE) producing sticky ends, then double stranded adapters (vectorettes) matching the overhangs but showing some internal mismatch ("bubble") are ligated. Using one primer specific to the sequence in question and the other specific to the reverse complement of one of the vectorette strands (in the region of mismatch), it is possible to directionally amplify the genomic fragment located between the specific primer and the RE recognition site. Multiple REs are usually used to ensure a fragment of sufficient length will be obtained. A nested approach, using an internal specific primer in the second PCR, facilitates elimination of false positives, i.e. spurious bands not representing the region of interest. Nested PCR was used in all the vectorette PCR experiments.

We adopted the modified vectorette PCR protocol of Ko et al. [65]. Briefly, ca. 3 μg portions of genomic DNA were digested with 20 U of *Bsu*15I, *Eco*RI, *Mun*I and *Xap*I REs (Fermentas) at 37°C for 4 hours in 100 μl volumes. Double stranded vectorette adapters consisting of vect53 and vect57GC for *Bsu*15I or vect57TTAA for the remaining REs [65] were ligated to digested DNA with 2000 U of T4 DNA ligase (New England Biolabs). The mixture was incubated overnight at 16°C. The ligase was inactivated by incubation of mixture at 65°C for 20 min. For the first vectorette PCR, we used a specific forward primer for amplification of the 3' portion of the 2^nd ^MHC class IIB exon and a reverse primer for the 5' portion. The 20 μl PCR reactions contained 10 μl of 2 × Hot Start Master Mix (Qiagen), 1 μM of the specific (FicLw1/FicRw1) and C20 vectorette [65] primers and 1 μl of vectorette-ligated digested DNA. The touchdown PCR scheme was as follows: 95°C/15 min., 5×(94°C/30 s, 64°C/30 s, 72°C/60 s), 5×(94°C/30 s, 60°C/30 s, 72°C/60 s), 20× (94°C/30 s, 58°C/30 s, 72°C/60 s), 72°C/3 min. In the second PCR nested forward (FicLw2, for 3') and reverse (FicRw2, for 5' end amplification) primers were used together with the B21 vectorette primer [65]. As template we used 0.5 μl of the product of the first PCR reaction; 20 μl of PCR mixture contained 2 μL of 10× PCR buffer with (NH_4_)_2_SO_4_, 2 mM MgCl_2, _1 μM of each primer, 0.2 mM of each dNTP and 1 U of *Taq *polymerase (Fermentas). The cycling scheme was as follows: 94°C/3 min, 30×(94°C/30 s, 58°C/30 s, 72°C/60 s), 72°C/3 min. The PCR product was run on a 1.5% agarose gel, clearly visible bands were excised, purified using the MinElute Gel Purification Kit (Qiagen) and directly sequenced with the nested specific and vectorette D19 [65] primer using the Big Dye Terminator (BDT) 3.1 chemistry (ABI). Sequencing reaction products were electrophoresed on an ABI 3130×l Genetic Analyzer. We used the consensus from the obtained sequences as well as sequences from other passerines to design a pair of specific primers within the second exon (FicL1938, FicR1938) which were used for further genotyping.

### cDNA analysis

To evaluate the expression status of MHC class IIB sequences obtained with FicL1938, FicR1938 primers we amplified, cloned and sequenced cDNA. Bursa Fabrici was extracted from three chicks from Niepołomice Forest (S Poland), preserved in the RNAlater reagent (Qiagen) and RNA was extracted with the RNeasy kit (Qiagen) including the DNAse treatment step to remove any DNA contamination. RNA was reverse transcribed to cDNA using Omniscript Reverse Transcriptase kit (QIAGEN) and Oligo(dT)_12-18 _primer (Invitrogen). cDNA was used as a template in PCR reactions with sequence-tagged FicL1938, FicR1938 primers (see below). PCR product for three individuals was purified with the MinElute PCR Purification Kit (QIAGEN) and T/A cloned using the Strataclone PCR cloning kit (Stratagene). Recombinant clones were detected by blue/white screening and inserts were directly amplified with M13F and M13R primers in colony PCR. Thirty two clones containing inserts were sequenced using Big Dye Terminator 3.1 chemistry on an ABI 3130×l genetic analyzer. Sequences were assigned to individuals on the basis of tag sequences (see below), checked and aligned in SeqScape 2.5 (ABI).

### Generation of amplicons and 454 sequencing

A 198-bp (without primers) fragment of the collared flycatcher MHC class IIB 2nd exon was amplified using fusion primers containing sequences of the primers FicR1938 and FicL1938 identified as described above. The forward fusion primer 5'-GCCTCCCTCGCGCCATCAGNNNNNNGAGTGTCHYTTCVTTAACGGCAC-3' was composed of the 454 FLX amplicon A primer, a 6-bp tag (indicated with Ns), used to distinguish individuals and the FicL1938 sequence (underlined); the reverse fusion primer consisted of the 454 FLX amplicon B primer and the FicR1938 sequence (underlined) 5'-GCCTTGCCAGCCCGCTCAGCTCTGCGCTCCACGVBGAACGGG-3'. Polymerase chain reaction was performed in 20 μl and contained approximately 100 ng of genomic DNA, 2 uL of 10× PCR buffer with (NH_4_)_2_SO_4_, 2 mM MgCl_2, _1 μM of each primer, 0.2 mM of each dNTP and 1 U of *Taq *polymerase (Fermentas). PCR scheme was: 94°C/3 min, 33×(94°C/30 s, 58°C/30 s, 72°C/30 s), 72°C/3 min. The concentration of the PCR product was estimated through agarose gel electrophoresis, and PCR products were pooled into approximately equimolar quantities. The resulting pools were purified using the MinElute PCR Purification Kit (QIAGEN). Purified pools were then sequenced as a part of a single 454 FLX run (the run contained also MHC amplicons from other species) according to the 454 Amplicon Sequencing protocols provided by the manufacturer (Roche 454) at the Functional Genomics Center, Uni ⁄ETH Zurich. Since only 35 tagged primers were used, the picotiter plate was divided into eight sections. The sequence determination was made using a GS Run Processor in the Roche 454 Genome Sequencer FLX Software Package 2.0.00.22. The performance of the sequencing run was gauged using known pieces of DNA introduced in the sequencing run as DNA Control Beads. On average, 95% of reads from DNA Control Beads matched the corresponding known sequences with at least 98% accuracy over the first 200 bases, which was above the typical threshold (80% matches of 98% accuracy over 200 bases).

Initially, reads containing FicL1938 and reverse complement FicR1938 sequences were extracted from the multifasta files and assigned to the respective individuals on the basis of the tag sequence. However, this procedure excluded ca. 15% of otherwise good reads which did not reach into the reverse primer. Therefore, finally we decided to analyze only first 188 bp of each read following the forward primer, which maximized the sequence yield while reducing the length of the analyzed fragment by only 5%. Extraction of sequences, assignment of reads to individuals and generation of alignments of variants present in each amplicon was performed with the custom software written in Java and available from http://code.google.com/p/jmhc/. Outputs from jMHC were further analysed in Excel.

### Distinguishing putative alleles from artifacts

For the initial assessment of sequences diversity we included all sequence variants which occurred in at least two independent PCR reactions, in each represented by at least three reads. The two PCR criterion is a standard in MHC studies [38], whereas the requirement of three copies in each PCR stems from the probabilistic model of Galan et al. [41], which showed that the probability of observing three times the same artifactual variant as a result of sequencing error is negligibly low. This heuristic 2-PCRs-3-copies-in-each criterion was useful for revealing general patterns of sequence diversity and relationships, while excluding most sequencing errors. However, PCR recombinants (chimeras) could still be present among variants which passed the 2-PCRs-3-copies-in-each threshold. Identical chimeras may be produced repeatedly during PCR by recombination between pairs of true alleles. Distinct recombination events may produce identical chimeras, thus they may be represented by a substantial number of sequencing reads derived from multiple amplicons. Some true alleles may have sequences identical to chimeras because of their evolutionary origin through an in vivo recombination between alleles already present in the population. However, a critical feature distinguishing true recombinants from PCR chimeras is that the latter should always co-occur with both parental sequences in the same amplicon, whereas true recombinants may or may not co-occur with one or both parental sequences. Using this rationale we applied the following procedure to distinguish putative expressed alleles (PEA) from PCR chimeras on the level of the whole dataset. We first calculated maximum per amplicon frequency (MPAF) for each putative expressed sequence variant which passed the 2-PCRs-3-copies-in-each threshold. Then we sorted the variants according to their MPAF. The variants were then examined starting from the lowest MPAF value as follows:

• we selected three amplicons in which the given variant was most abundant (including the one on which the MPAF value was based)

• we checked whether in all three amplicons the variant can be explained as a chimera of more abundant variants within the same amplicon - if so, the variant was classified as PCR chimera, otherwise it was classified as putative expressed allele (PEA).

Working from the bottom of the list (lowest MPAF = 0.51%) up, all variants with MPAF lower than 1.5% were checked. None of the variants with MPAF ≥ 0.97% was classified as PCR chimera. Among 10 randomly selected variants with MPAF > 1.5%, none could be classified as PCR chimera either. Therefore, we can safely assume that virtually all variants with MPAF ≥ 0.97% are PEA. We suppose that the apparent success of the above approach in identifying threshold frequency above which artifacts are absent results from the high average divergence among PEA (in contrast to pseudogene sequences, see below).

Below the threshold of 0.97%, however, both PCR chimeras and apparent PEA occur. Among 31 variants in this "grey zone", 11 could be classified as PCR chimeras. Thus, in the total sample of 159 variants, these 11 PCR chimeras constituted 6.9% of all PEA which passed the 2-PCRs-3-copies-in-each threshold. Additionally, two variants had one bp indel in homopolymer runs and were classified as sequencing artifacts.

Sequence variants within each pseudogene cluster were very similar to each other. Therefore, the proportion of variants which could have been explained as PCR chimeras was high and we could not observe a clear MPAF threshold above which chimeras could be excluded, as was the case in PEA - the "grey zone" in which apparent true pseudogene alleles and PCR chimeras co-occurred was very wide. Therefore, we decided to use the threshold we calculated for PEA, after adjusting for relative frequencies of expressed and pseudogene sequence variants among 454 reads. This assumes that the actual frequency of PCR recombination is similar for all variants.

The average per amplicon proportion of reads from PEA, cluster I and group II.A pseudogene PA were 0.164, 0.718 and 0.118, respectively. Based on the MAFT threshold distinguishing PEA from the "grey zone" (0.97%, se above), we computed thresholds for cluster I pseudogenes (4.25%) and group II.A pseudogenes (0.70%). In both pseudogene clusters we confirmed the occurrence of variants which could not have been explained as PCR chimeras below these thresholds. However, we chose not to explore this zone systematically, because given high similarity of pseudogene sequences, distinguishing PCR chimeras from true alleles would be unreliable. Including in the analysis only the pseudogene variants with MAFT above the grey zone threshold is certainly conservative and reduces the number of variants in pseudogene cluster I from above 225 to 28. It should be emphasized though, that this conservative approach does not change the general conclusion of high similarity among variants in pseudogene clusters. On the other hand, the minimum number of pseudogene loci obtained with this approach is certainly an underestimate. Nevertheless, we preferred this underestimation over the alternative of inflating the estimates of the number of loci by including artifacts.

### Relationships among the sequences

The relationships among the collared flycatcher's putative MHC class IIB alleles were reconstructed with a Neighbor Joining tree constructed from the matrix of Tamura-Nei nucleotide distances. The robustness of the topology was tested with 1,000 bootstrap replicates; the tree was rooted with the chicken sequence.

To place the collared flycatcher's MHC IIB sequences in the context of MHC IIB variation in Passeriformes, we downloaded the passerine sequences available from GenBank and performed the phylogenetic reconstruction together with a set of collared flycatcher sequences selected to represent the detected diversity. Selected MHC IIB sequences from all passerine species, which spanned the length of the fragment available for the collared flycatchers, were included in the tree. In case of the *Luscinia *sequences [35], which were slightly shorter than the collared flycatcher sequences, we filled in the missing parts with amplification primer sequences in order to analyze the full alignment length. Two Bayesian analyses under the GTR + Γ model of sequence evolution were run for 2.5 × 10^7 ^generations and sampled every 5,000 generations. The first 1,000 trees from each analysis were discarded as burn-in, resulting in 8,000 sampled trees used to calculate the posterior probability (PP) of each bipartition. Chicken and caiman MHC class IIB sequences were used as outgroups to root the tree.

### Detecting signatures of historical selection

The average rates of synonymous (dS) and nonsynonymous (dN) substitutions were computed for all sites, as well as for positions encoding amino acids forming the antigen-binding groove (Antigen Binding Sites, ABS), and positions encoding the remaining amino-acid (non-ABS) The location of ABS was inferred from the human MHC II molecular structure [66]. Computations were performed in MEGA4 [67].

The impact of historical selection on the MHC sequences was assessed through the Z-test of selection in MEGA and by fitting three models of codon evolution available in PAML [68]. These were: (i) M0: one ω (dN/dS ratio), (ii) M7: nearly neutral (ω ≤ 1) with the beta distribution approximating ω variation, and (iii) M8: positive selection (a proportion of sites evolving with ω > 1) with the beta distribution approximating ω variation. The best-fitting models were chosen on the basis of the value of the Akaike information criterion (AIC; [69,70]). Positively selected codons were identified through the Bayes empirical Bayes procedure [71].

### Recombination detection

We checked for signatures of recombination in our data set using three methods. Two of these, GeneConv [72] and MaxChi2 [73] performed very well in an assessment of 14 recombination detection methods [74]. They are implemented in the rdp3 software [75], used for computations. Additionally, a new method, genetic algorithm recombination detection (GARD; [76]), was applied, through a web-based routine http://www.datamonkey.org/.

Because the exploratory searches for recombination signals require repetitive statistical testing, a multiple test correction is necessary. As the number of tests increases exponentially with the number of sequences, taking into account the very high number of putative alleles detected in our experiment, tests for recombination using all sequences would be extremely conservative due to the severity of multiple comparison correction. Therefore we used two complementary approaches to detect recombination. First, we selected from the dataset 25 representative sequences, including these which formed separate long branches, as they might have been recombinants, second we randomly chosen ten sets of 25 sequences and performed on them the same analyses. In our opinion such approach is a reasonable way to qualitatively assess the signals of recombination in our dataset.

## Authors' contributions

MZN participated in study design, carried out the laboratory analyses and helped to draft the manuscript, WB analyzed the data and drafted the manuscript, MS provided tools for bioinformatic analyses, LG provided the samples, MC participated in study design and coordination, JR participated in study design and coordination and helped to draft the manuscript. All authors read and approved the final manuscript.
